# Evolutionary dynamics of HIV-1 subtype C accessory and regulatory genes in primary infection

**DOI:** 10.1186/1742-4690-9-S2-P142

**Published:** 2012-09-13

**Authors:** R Rossenkhan, V Novitsky, TK Sebunya, R Musonda, BA Gashe, M Essex

**Affiliations:** 1 HSPH/UB/BHP, Boston, MA, Botswana; 2 Harvard School of Public Health (HSPH)/ BHP, Boston, MA, USA; 3 University of Botswana (UB), Gaborone, Botswana; 4 Botswana Harvard AIDS Institute Partnership (BHP), Gaborone, Botswana

## Background

Studies addressing the dynamics of accessory and regulatory viral gene diversity and selection during early stage of HIV-1 infection are limited but crucial for progress towards vaccine research.

## Methods

Intra-patient diversity and evolution was assessed during primary HIV-1C infection, viral quasispecies were obtained by single genome amplification (SGA) at multiple sampling time points up to one year post-seroconversion (p/s).

## Results

The mean intra-patient diversity was found to be 0.11% (95%CI; 0.02 to 0.20) for vif, 0.23% (95%CI; 0.08 to 0.38) for vpr, 0.35% (95%CI; -0.05 to 0.75) for vpu, 0.18%(95%CI; 0.01 to 0.35 ) for tat exon 1 and 0.30% (95%CI; 0.02 to 0.58) for rev exon 1 during the time period 0 to 90 days p/s. The intra-patient diversity increased gradually in all non-structural genes over the first year of HIV-1 infection, which was evident from the vif mean intra-patient diversity of 0.46% (95%CI; 0.28 to 0.64), vpr 0.44% (95%CI; 0.24 to 0.64), vpu 0.84% (95%CI; 0.55 to 1.13), tat exon 1 0.35% (95%CI; 0.14 to 0.56 ) and 0.42% (95%CI; 0.18 to 0.66) for rev exon 1 during the time period of 181 to 500 days p/s. Statistically significant increases in viral diversity were observed for vif (p=0.013) and vpu (p=0.002). Weak and sporadic associations between levels of viral diversity within the non-structural genes and HIV-1 RNA load during primary infection were found. Positive and negative selection patterns over the first year post-seroconversion were assessed in each of these genes, providing insight into the selection pressures on these genes which are crucial for viral replication in-vivo.

## Conclusion

Our study highlights differential diversity and slower diversification across these HIV-1 genes. The most likely cause is different selection pressure imposed by host immune response to the encoded viral gene products that may result in different evolutionary rates.

